# Effect of bleaching agents on sealing properties of different intraorifice barriers and root filling materials

**DOI:** 10.4317/medoral.17751

**Published:** 2012-02-09

**Authors:** Ebru Canoglu, Kamran Gulsahi, Cem Sahin, Emre Altundasar, Zafer C. Cehreli

**Affiliations:** 1 DDS. Research Assistant, Department of Pediatric Dentistry, Faculty of Dentistry, Hacettepe University, Ankara, Turkey; 2 DDS, PhD. Assistant Professor, Department of Endodontics, Faculty of Dentistry, Baskent University, Ankara, Turkey; 3 DDS, PhD. Research Associate, Department of Prosthetic Dentistry, Faculty of Dentistry, Hacettepe University, Ankara, Turkey; 4 DDS, PhD. Research Associate, Department of Endodontics, Faculty of Dentistry, Hacettepe University, Ankara, Turkey; 5 DDS, PhD. Professor, Department of Pediatric Dentistry, Faculty of Dentistry, Hacettepe University, Ankara, Turkey

## Abstract

Objective: To evaluate the effect of intracoronal bleaching agents on the sealing properties of different intraorifice barriers and root filling materials. Study Design: The root canals of extracted human premolars (n=180) were prepared by using System GT rotary files and filled with either gutta-percha+AH Plus or Resilon+Epiphany sealer. In both groups, the coronal 3mm of root filling was removed and replaced with one of the following materials applied as intraorifice barriers (n=30/group): 1. ProProot-MTA; 2. Conventional Glass ionomer cement; and 3. Hybrid resin composite. In each subgroup, intracoronal bleaching was performed using either sodium perborate with distilled water or 35% hydrogen peroxide gel for 3 weeks. The leakage of specimens was measured using fluid-filtration and dye penetration tests. The data were analyzed statistically with One-way ANOVA, Repeated Measures t-test and Independent Samples t-test (p=0.05). Results: The fluid conductance values of the test groups were not influenced by the type of the bleaching agent, the intraorifice barrier, or the root filling material (all p>0.05). However, the extent of dye leakage was significantly affected by the type of intraorifice barrier material (p<0.05), which showed the following statistical ranking: glass ionomer cement > resin composite > ProRoot-MTA (p<0.05). Conclusions: The effect of 35% hydrogen peroxide gel or sodium perborate/distilled water on the sealing properties of tested intraorifice barriers and root filling materials varied conforming leakage assessment. These properties were not affected by using fluid filtration test, while the glass ionomer barrier showed the greatest amount of dye leakage in both gutta-percha and Resilon root-filled teeth.

** Key words:**Tooth Bleaching, root canal filling materials, glass ionomer cement, mineral trioxide aggregate, micro leakage

## Introduction

Ideally, the root canal filling material should provide a barrier by itself that prevents bacterial ingress from the oral cavity and travelling down the root canal and to periapical tissues (1). However, none of the current obturation materials/techniques are capable of providing such a level of seal. Both gutta-percha, in combination with sealers, and the relatively newer adhesive obturation systems such as Resilon and Epiphany fail to prevent leakage effectively within the root canal system (2). This problem has led to the recommendation that core materials be placed at the orifice of the root canal directly after the completion of orthograde root canal treatment (3).

Placement of a reliable intraorifice barrier is generally required during intracoronal bleaching of root-filled teeth. Hydrogen peroxide is the active ingredient of contemporary bleaching materials, which can be applied directly or can be produced by a chemical reaction from carbamide peroxide or sodium per borate (4). Because of its low molecular weight, hydrogen peroxide can penetrate the dentin and release oxygen that breaks the double bonds of the organic and inorganic compounds inside the dentinal tubules (5). Despite the lack of direct evidence, diffusion of bleaching agents through dentin tubules into periodontal tissues has often been associated with invasive cervical root resorption, the most dramatic adverse effect of the intracoronal bleaching technique (6,7). Thus, sealing the root canal orifice appears to be essential to prevent diffusion of bleaching agents from the pulpal chamber into the root canal and the cervical periodontal tissue (5,8,9). On the other hand, the sealing properties of restorative materials used as intraorifice barriers may be jeopardized by the negative effects of bleaching agents including changes in their chemical and physical properties (10). Because the severity of these effects could depend both on the bleaching agent (10) or the type of restorative material used (11,12), it is essential to evaluate the effects of non-vital bleaching agents on different intracoronal barrier materials.

Methods utilized for leakage assessment during intracoronal bleaching include dye penetration (13,14), fluid filtration (15), chemical (16) and microbial (16,17) tests. Thus, this study utilized fluid filtration and dye penetration tests to evaluate the effect of different bleaching agents on the sealing properties of different intraorifice barriers and root filling materials. The study tested the threefold null hypothesis that the type of (1) bleaching agent, (2) intraorifice barrier, or (3) root filling material does not affect the intracoronal sealing ability as measured by the two aforementioned leakage tests.

## Material and Methods

Tooth selection

Freshly-extracted, human mandibular premolars were selected on the basis of their macroscopically similar size and straight roots, and stored in an aqueous solution of 0.5% chloramine-T at 4°C until experiments (a maximum of 1 month). The crowns were sectioned off below the cementoenamel junction, so that the length of all roots was adjusted to approximately 16 mm. Thereafter, all specimens were examined under a stereo microscope (40X) to ensure the absence of cracks, leaving a total of 180 roots available for experimental procedures.

Specimen preparation

The canal length was determined with a #10 K-file, and the working length was determined by subtracting 1 mm from the canal length. The roots canals were instrumented with 0.4 taper System GT rotary files (Dentsply Tulsa Dental, Tulsa, OK, USA) in conjunction with RC-Prep lubrication (Premier Dental Products, Tulsa, OK, USA) and 2 mL of 5.25% sodium hypo chlorite (NaOCl) irrigation between each file size. All canals were enlarged to ISO #40 to the working length. The root canals received a final irrigation of 5 mL 17% ethylenediaminetetraacetic acid (EDTA) and 5 mL 5.25% NaOCl, after which the canals were flushed with 10 mL distilled water and dried with paper points.

The specimens were randomly assigned into two groups with respect to the root filling material used (n=90/group): Group I: Gutta-percha+AH Plus sealer (Dentsply Caulk, Milford, DE, USA) and Group II: Resilon+Epiphany Sealer (Pentron Clinical Technologies, Wallingford, CT, USA). In both groups, #40 0.4-taper single cones were used. The sealers were prepared and applied according to the manufacturer’s directions. In group II, the specimens were light-cured from the coronal aspect using a quartz-tungsten-halogen unit for 40s. Following obturation procedures, approximately 3 mm of root fillings were meticulously removed from their corona aspect (18) with the aid of heated instruments and 70% alcohol-moistened micro brushes so as to prepare space for intraorifice barriers. In both obturation groups, specimens were further subgrouped with respect to the intraorifice barrier material placed over root fillings: (1) ProRoot-MTA, (Dentsply Tulsa Dental, Tulsa, OK, USA), (2) Conventional glass ionomer cement (Fuji Triage, GC Corp, Tokyo, Japan), and (3) Hybrid resin composite (TPH Spectrum, Dentsply/Caulk) in conjunction with a total-etch dentin bonding agent (XP Bond, Dentsply/Caulk). The test materials were prepared and applied in strict accordance with the manufacturers’ instructions. All materials were flush with the sectioned root surface. The specimens were stored at 37°C and 100% humidity for 1 week to allow the materials to set completely.

Bleaching procedures

10 mm-long plastic rings were tightly adapted on the coronal portion of roots to serve as a bleaching chamber. Two bleaching agents were tested: sodium perborate and 35% hydrogen peroxide. Sodium per borate tetrahydrate (Sigma-Aldrich, St. Louis, MO, USA) was combined with a vehicle (distilled water) to maintain a 2:1 ratio in solution (about 0.1 g sodium per borate and 0.05 ml distilled water) (19). 35% hydrogen peroxide was tested in the form of a commercial bleaching gel (Opalescence Endo, Ultradent, South Jordan, UT, USA). In both groups, 0.1g of the bleaching agent was placed into the bleaching chamber, after which the chamber was sealed with a temporary material (Cavit, 3M ESPE, Seefeld, Germany). After 7 days, Cavit was removed and the bleaching agent was washed out with air-water jet for 60s. Thereafter, a fresh portion of the bleaching agent was placed into the chamber. This procedure was repeated every 7 days for 3 weeks, in accordance with the walking bleach technique (19). In the control (no bleaching) groups, a cotton pellet soaked with distilled water was placed into the chamber and replaced every 3 weeks (19). During the bleaching procedures, the specimens were kept in an incubator at 37°C, wrapped in gauze soaked with distilled water.

Evaluation of Leakage

A modified fluid transport test was used to measure apical leakage (18). The leakage was quantified by following the movement of a tiny air bubble traveling within a constant bore a 100-µL micro pipette. All pipettes, syringes, and plastic tubes used in the system were filled with deionized water. A micro pipette was connected to the plastic tube, and this tube was connected to the root with epoxy resin (Pattex; Henkel, Düsseldorf, Germany). Water was drawn back approximately 2 mm with the micro syringe to introduce a tiny air bubble in the micro pipette. The air bubble was adjusted to a suitable position within the micro pipette with the syringe. Finally, regulated air from a pressure tank at 121.6 KPa (1,240 cmH2O) (18) was applied from the coronal parts of the specimens, forcing water through any voids along the root canal filling. The water movement displacing the air bubble in the capillary tube was measured per unit of time. Linear displacement of this air bubble was converted to volume displacement and was recorded as the fluid transported. The values were expressed as µL/min/cm H2O. For specimens serving as the positive control, procedures for selection and instrumentation were the same as those described for the experimental groups, except that the prepared root canal space was not obturated. The fluid flow rate through the unfilled root canal was measured by recording the movement of the air bubble that could pass through the root canal in 1 minute (1428 µL/min/cm H2O). This value served both as a positive control and as 100% leakage, to which the sealed values could be compared. The leakage values were measured by the fluid-filtration method at days 1 and 7. Except for the fluid-filtration measurements, the specimens were stored in 100% humidity during the entire experimental period.

Micro leakage Evaluation

Following fluid filtration procedures, the specimens were assigned for the assessment of intracoronal dye leakage. The apices were sealed with sticky wax, after which the root surfaces were coated with two consecutive layers of nail varnish up to 1 mm from the coronal barrier margins. Samples were then immersed in 0.5% basic fuchsin solution at 37°C for 24h. Thereafter, specimens were rinsed thoroughly under tap water, and the nail varnish was removed with a scalpel. The roots were sectioned longitudinally in the buccolingual direction, and a digital photograph of each section was obtained at 20X under a stereomicroscope (Olympus; Tokyo, Japan). The images were transferred to a Macintosh PowerPC workstation, and the extent of dye penetration (from coronal to the most apical dye mark) (20) was measured (in mm) on digital images by using image analysis software (ImageJ for MacOSX; v.1.34, National Institutes of Health; Bethesda, MD, USA).

Statistical Analysis

The fluid conductance and dye penetration data were analyzed with SPSS statistical software, version 13.0 (SPSS Inc, Chicago, IL, USA). For the fluid filtration measurements, One-way ANOVA was used to determine the differences within and between the experimental and the control groups, respectively (p=0.05). Statistical comparisons between the 24h and one-week fluid conductance values were made with Repeated Measures t-test (p=0.05).

One-way ANOVA and Student-Newman-Keuls tests were used to determine statistical differences between the dye penetration values of the experimental and control groups (both p=0.05). Independent Samples t-test was used to compare the dye leakage values with respect to the root filling materials used (p=0.05).

## Results

The fluid conductance values of the experimental (bleaching) and control groups at 24h and 1 week are presented in ( Table 1) as mean±standard deviation. There were no significant differences among the experimental groups, among the control groups, and between the experimental and control groups (One-Way ANOVA, all p>0.05). Similarly, no significant differences were found between the 24h and 1-week fluid conductance values of the experimental or control groups. (Repeated Measures t-test, p>0.05).

**Table 1:**
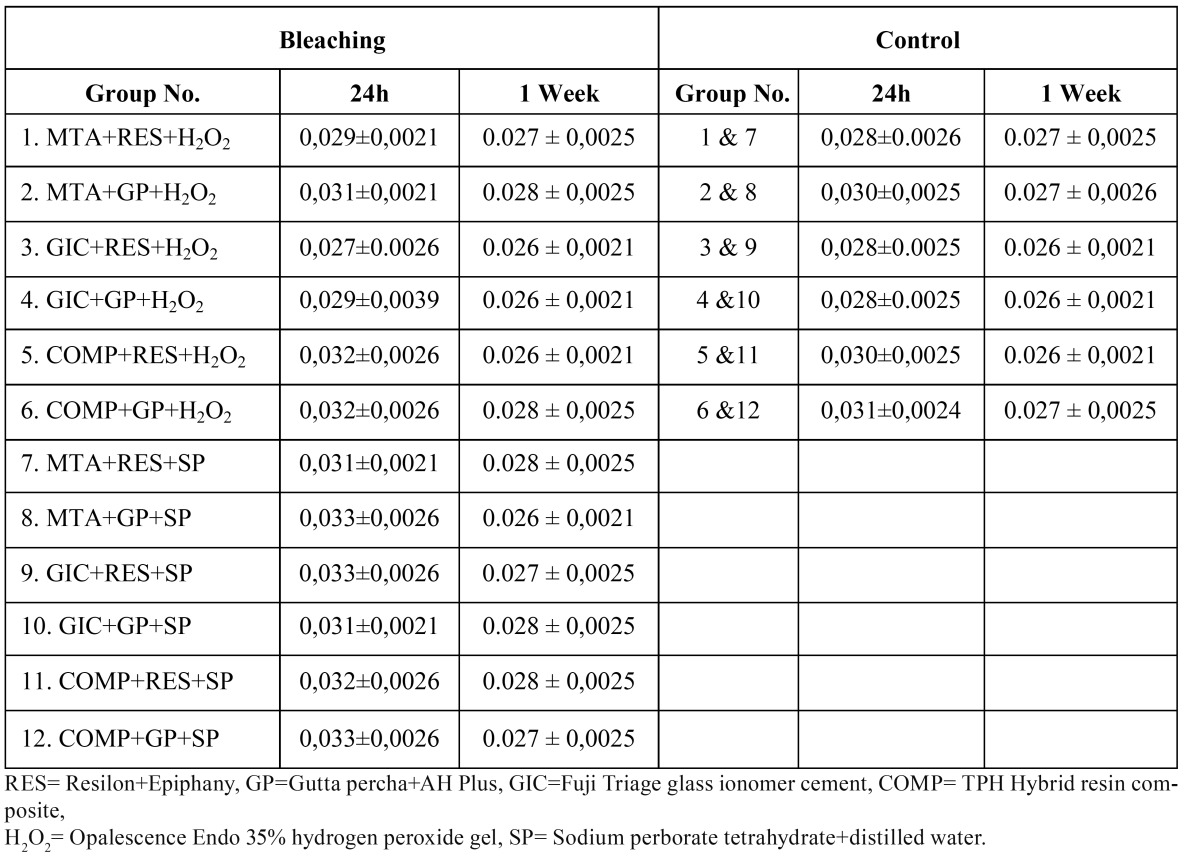
Fluid conductance values (mean±SD) of the experimental and control groups at 24h and 1 week. The values are expressed in µL/min/cm H2Ox10-5.

The coronal dye penetration values (mm) are presented in ( Table 2) as mean±standard deviation. Irrespective of the intraorifice barrier and/or root filling material used, both hydrogen peroxide gel and sodium perborate with distilled water yielded similar amounts of dye leakage (Independent samples t-test, p>0,05), and these values did not differ significantly from their respective control (no bleaching) groups (One-way ANOVA, p>0.05).

**Table 2:**
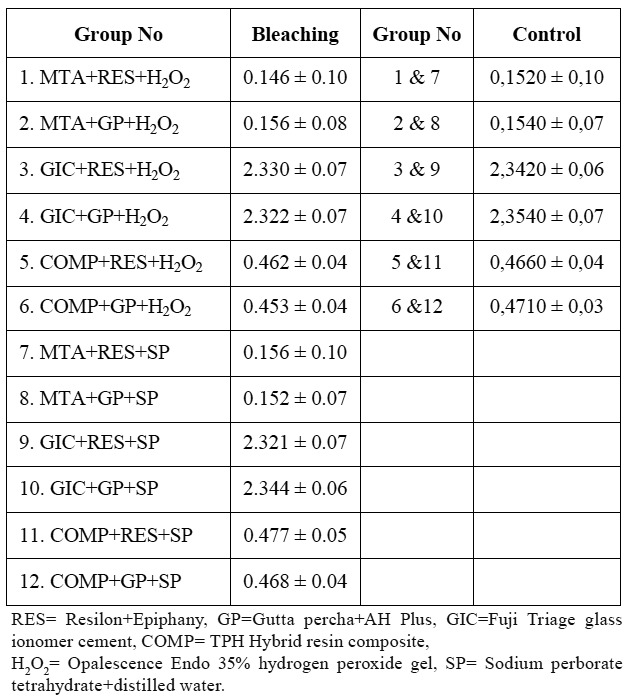
The extent of coronal dye leakage in the experimental and control groups. The values (mm) are expressed as mean±SD.

A comparison of dye penetration values within both the experimental and control groups showed that dye leakage was significantly affected by the type of intraorifice barrier material (One-way ANOVA, p<0.05). Accordingly, in both the experimental and control groups, the use of Fuji Triage glass ionomer cement as an intraorifice barrier resulted in the greatest amount of dye penetration (Student-Newman-Keuls test, p<0.05).

The following statistical ranking was observed with regard to the amount of leakage (in mm): glass ionomer cement> resin composite> ProRoot-MTA.

With regard to the effect of root filling materials on dye leakage, no comparison could be made in the MTA and resin composite groups, as the level of dye penetration did not exceed the apical level of those barrier materials. In the glass ionomer groups, the dye leakage extended below the intraorifice barrier, but showed similar levels of penetration in the gutta-percha and Resilon groups (Independent samples t-test, p>0.05).

## Discussion

The aim of this study was to investigate whether intracoronal bleaching with 35% hydrogen peroxide gel or sodium per borate with distilled water would influence the extent of fluid conductance and dye penetration of different intraorifice barriers and root filling materials. Although a bacterial leakage model may appear to be more clinically relevant compared with a fluid filtration model, the latter technique was used herein because it is currently the only nondestructive, quantitative testing method that enables measurement of micro leakage from the same specimens at intervals over extended periods (21). The dye penetration test was used in conjunction with the fluid filtration model, as it allows assessment of regional (in this case, coronal) micro leakage (22).

The present results showed that intracoronal bleaching with 35% hydrogen peroxide gel and sodium per borate with distilled water did not alter the fluid conductance values in comparison with their respective control groups. Since results of the dye penetration tests also yielded similar findings, it can be concluded that the tested bleaching agents do not jeopardize the sealing properties of the intraorifice barrier materials, leading to acceptance of the first null hypothesis. The fact that the bleaching agents did not cause further micro leakage may possibly be due to their unstable chemical nature that is insufficient to disrupt the dentin-intraorifice barrier interface during the three successive weekly bleaching episodes (5,23). Thorough removal of the residual bleaching material after each weekly application may also have contributed to the diminished adverse chemical effect of hydrogen peroxide on dentin, which might render the tooth-barrier interface prone to micro leakage (5,24).

According to Wolcott et al (17), ideal properties of an intraorifice barrier should include the following characteristics: 1. easily placed, 2. bonds to tooth structure, 3. seals against micro leakage, 4. distinguishable from natural tooth structure, and 5. does not interfere with the final restoration. Fuji Triage is a pink-colored glass ionomer cement which satisfies the five criteria proposed for an ideal intraorifice barrier (17). The effectiveness of Fuji Triage as an intraorifice barrier has been demonstrated previously (25). Moreover, the sealing efficiency of Fuji Triage and a similar glass ionomer, Fuji II LC, have been found to be analogous to those of grey and white MTA intraorifice barriers, as verified with the bacterial leakage (26) and fluid transport (27) models, respectively. However, when a dye penetration method was employed, it was found that even a 4mm-thick glass-ionomer intraorifice barrier leaked more than MTA (14). The present results corroborate with those of the latter study, demonstrating that Fuji Triage displayed significantly less sealing capacity compared with MTA and the hybrid resin composite barriers. This finding also justifies our attempt to utilize a conventional dye penetration test as a complementary tool to determine the individual sealing properties of the intraorifice barriers and root filling materials, which has not been possible through use of the fluid transport model. Thus, the differences in the extent of dye leakage between the intraorifice barrier materials lead to partial rejection of the second null hypothesis. Unlike the MTA and hybrid resin composite groups, the dye leakage extended below the glass ionomer barrier, but showed similar levels of penetration in the gutta-percha and Resilon groups. Consequently, the third null hypothesis that there are no differences in the coronal sealing ability of tested root filling materials should be accepted. According to the present results, the sealing performance of TPH is comparable to that of MTA, which is known to possess outstanding sealing properties. Unlike MTA, the placement of an acid-etch composite resin material is technically challenging, but offers the clinical advantage of an on-demand set, which enables subsequent application of the bleaching agent at the same appointment (28).

In both the experimental and control (no bleaching) groups, it was observed that placement of an intraorifice barrier over the root canal obturation did not completely prevent fluid conductance and dye leakage through the den tin-barrier interface. Undoubtedly, placement of such a cervical base will reduce apical leakage of the bleaching agent compared to canals without such a base (13,29). The present results also imply that MTA and the hybrid composite may retain their sealing capacity in the post-bleaching phase, which is crucial to minimize the adverse effects of the inevitable, long-term coronal micro leakage (23). However, the present experimental setup cannot determine if the bleaching agents penetrated into the dentin tubules from the canal wall towards the root surface. Thus, while ideal sealing of the root canal obturation is a critical aspect of preventing the side-effects of intracoronal bleaching, providing protection against external cervical resorption still appears to be a major concern that has to be resolved.

## Conclusion

The sealing properties of tested intraorifice barriers and root filling materials during intracoronal bleaching varied conforming leakage assessment. As verified with the fluid conductance test, the type of the bleaching agent (35% hydrogen peroxide gel or sodium per borate/distilled water) did not affect the sealing ability of the materials. However, glass ionomer barrier showed the greatest amount of dye leakage, followed by resin composite and ProRoot-MTA in both gutta-percha and Resilon root-filled teeth.
